# Growing connections: PlantConnectome maps molecular networks in Arabidopsis

**DOI:** 10.1093/plcell/koaf172

**Published:** 2025-07-02

**Authors:** Jan Wilhelm Huebbers

**Affiliations:** Assistant Features Editor, the Plant Cell, American Society of Plant Biologists; Unit of Plant Molecular Cell Biology, Institute for Biology I, RWTH Aachen University, Aachen 52056, Germany

One of the core strengths of large language models (LLMs) is their ability to reason across vast amounts of text, enabling them to extract functional relationships between entities and words. In essence, an LLM is like someone who has read millions of stories and can map how characters and ideas are connected based on how they appear together.

In new work, **Shan Chun Lim and colleagues (2025)** used this feature of LLMs to connect the dots between various entities in plant biology, including gene products, metabolites, tissues, and organs. The authors recognized that transforming the vast amount of unstructured information in the literature into a structured knowledge graph is one of the central challenges in biology. They also noted that the ever-growing body of literature makes it increasingly difficult to formulate strong hypotheses about functional biological relationships. The situation is much like solving a 100-piece versus a 1000-piece puzzle: while the larger puzzle provides a more detailed picture, it also takes considerably more time to complete. Computational tools can help solve the puzzle. Several such tools already exist, but many require laborious and inflexible preprocessing of input data, or they provide limited outputs (e.g. no gene–gene relationships or no information on interaction types), or both. To address these limitations, the authors used carefully fine-tuned prompts to text-mine over 71,000 plant research papers and compiled the functional relationships into a user-friendly database, **PlantConnectome**.

For their analysis, the authors focused on articles that mentioned *Arabidopsis thaliana* genes in their abstracts, totaling 71,136 articles, of which 19,809 were programmatically accessible as full texts. As a first step, they conducted a meta-analysis of the downloaded abstracts. This included an overview of the current plant literature landscape, revealing distinct groupings, for example, by experimental procedures, plant organs, and biological processes.

To text-mine the retrieved full-text articles and abstracts, they used OpenAI's ChatGPT models to identify (i) the functional relationships between pairs of entities (e.g. gene products, metabolites, tissues), (ii) the experimental basis for each relationship, and (iii) the definition of the extracted entities (Fig.). This pipeline ultimately yielded a knowledge graph representing 4.8 million functional relationships among more than 2.7 million entities.

**Figure.**
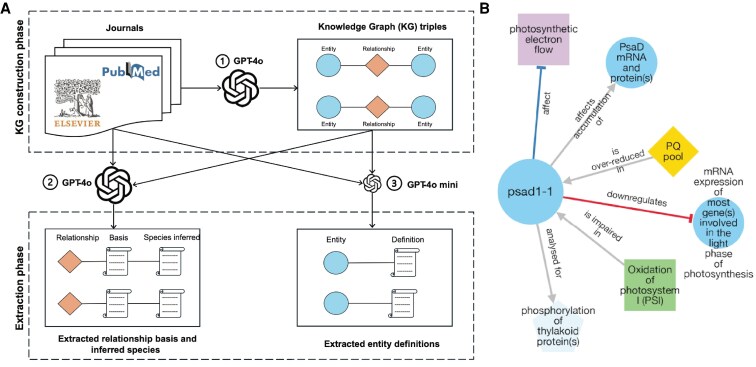
Abstract meta-analysis and knowledge graph visualization pipeline for 71,136 plant articles. **A)** Workflow illustrating the extraction of (1) the knowledge graph from the literature, (2) species-level relationships, and (3) entity definitions. Reprinted from Lim et al. (2025), Figure 2. **B)** Knowledge graph for the *psad1-1* allele (*PSAD*: *Photosystem subunit D-1*, *AT4G02770*) as retrieved from PlantConnectome. Node shapes and colors represent different entity types, while edge styles indicate relationship types. The network is interactive and includes clickable elements.

The authors also acknowledged the limitations of LLMs, including their tendency to hallucinate and misinterpret text. For example, they manually evaluated a subset of extracted edges (i.e. relationships between 2 entities) and observed high accuracy of over 85%. Most remaining errors involved misinterpretation of hypotheses as facts, which could largely be addressed by fine-tuning models with manually curated input. They also observed that redundancies—such as the use of “Arabidopsis” versus “*A. thaliana*”—inflate the size of the central knowledge graph (Fig.). To address this, they implemented a protocol to resolve these synonym-related issues.

PlantConnectome is a community-driven effort designed to systematically advance plant biology. It builds on a carefully analyzed foundation of user needs and was crafted by developers of other web applications such as GeneCat (Mutwil et al. 2008), PlaNet (Mutwil et al. 2011), and CoNekT (Proost and Mutwil 2018). This study not only contributes a valuable tool to the field, but it also demonstrates what is possible when scientific research is accessible.

Like other machine learning approaches, the depth and accuracy of PlantConnectome's insights scale with the size and quality of its input data. In this release, 71,136 research articles were analyzed, but approximately 72% (51,327) were abstracts only. The full-text versions were either not published open access or were unavailable for high-throughput download. This limitation serves as a clear reminder of the importance of open science and raises the question: How much more powerful could tools like PlantConnectome be if research were truly open?

## Recent related articles in *The Plant Cell*:

- Grones et al. (2024) proposed general guidelines for executing single-cell transcriptomics and analyzing and storing the retrieved data.
- Lasky et al. (2023) utilized genotype–environment association analyses to uncover the molecular basis of environmental adaptation in *Arabidopsis thaliana* across diverse environmental gradients.
- VanBuren et al. (2022) presented “Plants & Python,” a series of lessons integrating plant biology and Python programming to enhance computational literacy in plant science education.
- Li et al. (2022) developed “LeafNet,” a deep learning–based tool for automated localization and quantification of stomata and pavement cells from diverse leaf epidermal micrographs.

## Data Availability

Not applicable.

## Dive Curated Terms

The following phenotypic, genotypic, and functional terms are of significance to the work described in this paper:

AT4G02770 Gramene: AT4G02770
AT4G02770 Araport: AT4G02770

PSAD Gramene: AT4G02770
PSAD Araport: AT4G02770
